# Supplementary material for: A three-electrode dual-power-supply electrochemical pumping system for fast and energy efficient lithium extraction and recovery from solutions
Source: Commun Eng. 2024 Feb 10;3:29. doi: 10.1038/s44172-024-00174-8 (PMC10955941; doi:10.1038/s44172-024-00174-8)
Supplement: Supplementary file 2 — Supplementary Information [file 44172_2024_174_MOESM2_ESM.pdf]

**Supplementary Information**

# A three-electrode dual-power-supply electrochemical pumping system for fast and energy efficient lithium extraction and recovery from solutions

Kazuya Sasaki[1,2], Kiyoto Shin-mura[1,2], Shunsuke Honda[1], Hirofumi Tazoe[3] & Eiki Niwa[1]

[1]Graduate School of Science and Technology, Hirosaki University, 3 Bunkyo-cho, Hirosaki, Aomori 036-8561, Japan

[2]Lithium Resources Research Organization, Hirosaki University, 3 Bunkyo-cho, Hirosaki, Aomori 036-8561, Japan

[3]Institute of Radiation Emergency Medicine, Hirosaki University, 61-1 Honmachi, Hirosaki, Aomori 036-8564, Japan

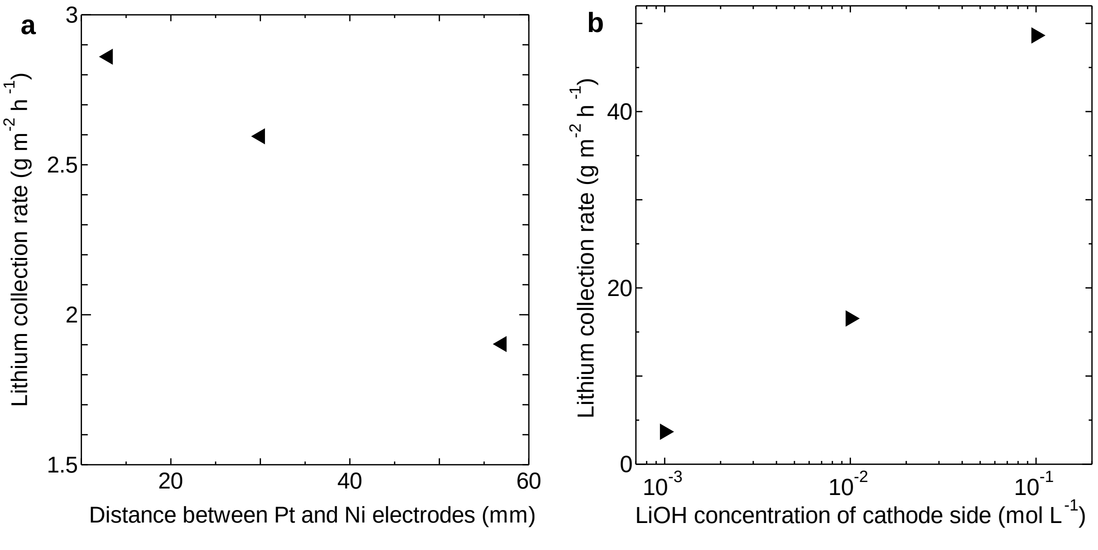


**Supplementary Fig. 1 |** Dependence of lithium collection rate on **a** distance between the

second and third electrodes (anode-side solution, 1.0 mol/L aqueous LiOH; cathode-side solution, $1 \times 10^{-3}$ mol/L aqueous LiOH), and **b** LiOH concentration of the cathode-side solution (anode-side solution, 1.0 mol/L aqueous LiOH; distance between second and third electrodes, 57 mm).
